# Comparing Catastrophic Costs: Active vs. Passive Tuberculosis Case Finding in Urban Vietnam

**DOI:** 10.3390/tropicalmed8090423

**Published:** 2023-08-23

**Authors:** Luong V. Dinh, Anja M. C. Wiemers, Rachel J. Forse, Yen T. H. Phan, Andrew J. Codlin, Kristi Sidney Annerstedt, Thuy T. T. Dong, Lan Nguyen, Thuong H. Pham, Lan H. Nguyen, Ha M. T. Dang, Mac H. Tuan, Phuc Thanh Le, Knut Lonnroth, Jacob Creswell, Amera Khan, Beatrice Kirubi, Hoa B. Nguyen, Nhung V. Nguyen, Luan N. Q. Vo

**Affiliations:** 1 National Lung Hospital, Ha Noi 10000, Vietnam; 2 Friends for International TB Relief, Ha Noi 10000, Vietnam; 3 WHO Collaboration Centre on Tuberculosis and Social Medicine, Department of Global Public Health, Karolinska Institutet, 171 76 Stockholm, Sweden; 4 Center for Development of Community Health Initiatives, Ha Noi 10000, Vietnam; 5 IRD VN, Ho Chi Minh City 700000, Vietnam; 6 Ha Noi Lung Hospital, Ha Noi 10000, Vietnam; 7 Pham Ngoc Thach Hospital, Ho Chi Minh City 700000, Vietnam; 8 Hai Phong Lung Hospital, Hai Phong 188140, Vietnam; 9 Da Nang Lung Hospital, Da Nang 550000, Vietnam; 10 Stop TB Partnership, Le Grand-Saconnex, 1218 Geneva, Switzerland

**Keywords:** active case finding, social protection, tuberculosis, catastrophic cost, out of pocket

## Abstract

Active case finding (ACF) is a strategy that aims to identify people with tuberculosis (TB) earlier in their disease. This outreach approach may lead to a reduction in catastrophic cost incurrence (costs exceeding 20% of annual household income), a main target of WHO’s End TB Strategy. Our study assessed the socio-economic impact of ACF by comparing patient costs in actively and passively detected people with TB. Longitudinal patient cost surveys were prospectively fielded for people with drug-sensitive pulmonary TB, with 105 detected through ACF and 107 passively detected. Data were collected in four Vietnamese cities between October 2020 and March 2022. ACF reduced pre-treatment (USD 10 vs. 101, *p* < 0.001) and treatment costs (USD 888 vs. 1213, *p* < 0.001) in TB-affected individuals. Furthermore, it reduced the occurrence of job loss (15.2% vs. 35.5%, *p* = 0.001) and use of coping strategies (28.6% vs. 45.7%, *p* = 0.004). However, catastrophic cost incurrence was high at 52.8% and did not differ between cohorts. ACF did not significantly decrease indirect costs, the largest contributor to catastrophic costs. ACF reduces costs but cannot sufficiently reduce the risk of catastrophic costs. As income loss is the largest driver of costs during TB treatment, social protection schemes need to be expanded.

## 1. Introduction

Tuberculosis (TB) remains a leading cause of death from an infectious disease [1]. A key step to reduce TB deaths and transmission is the closing of the treatment coverage gap. Globally, the difference between the estimated number of people who develop TB and those who are detected, treated, and notified to governments stands at 4.2 million people in 2021 [2,3]. Active case finding (ACF) is a strategy to identify people with TB in increased numbers and at an earlier stage of the disease generally by conducting screening activities closer to communities. When implemented in high TB burden populations, it can be a cost-effective intervention to reduce TB in the community [4,5,6]. Moreover, earlier diagnosis and treatment may reduce costs for affected individuals [7].

TB is closely linked with poverty. Socio-economic factors such as homelessness, overcrowded living spaces and malnutrition increase the risk of TB and negative treatment outcomes [8]. TB treatment costs, including out-of-pocket medical expenses and indirect costs due to income or job loss, lead to further impoverishment. Thus, eliminating catastrophic cost incurrence, defined as TB-related total patient costs exceeding 20% of the household’s annual income prior to the diagnosis, is a key target of the World Health Organization’s End TB Strategy [9]. However, half of all households affected by TB globally experience catastrophic cost [3]. In Vietnam, the rate is even higher, with 63% of households being affected [10].

The research on the economic benefits of ACF, though limited, is encouraging. Eight studies found a reduction in TB-related patient/household costs when comparing ACF with passive case finding (PCF). Five of six studies reported reductions in catastrophic cost, of which three were significant [7,11,12]. A study carried out in Ho Chi Minh City (HCMC), Vietnam’s largest city, prior to the COVID-19 pandemic found a significantly lower risk of catastrophic cost in persons detected through ACF [12]. These effects have not been investigated in other parts of the country.

The aim of our study was to assess whether ACF can decrease catastrophic cost incurrence, total TB-related costs, and socio-economic consequences in people with TB in the broader urban Vietnamese population.

## 2. Materials and Methods

This prospective cohort study collected longitudinal patient cost surveys in people with TB detected through ACF and PCF between October 2020 and March 2022.

**Figure 1:**
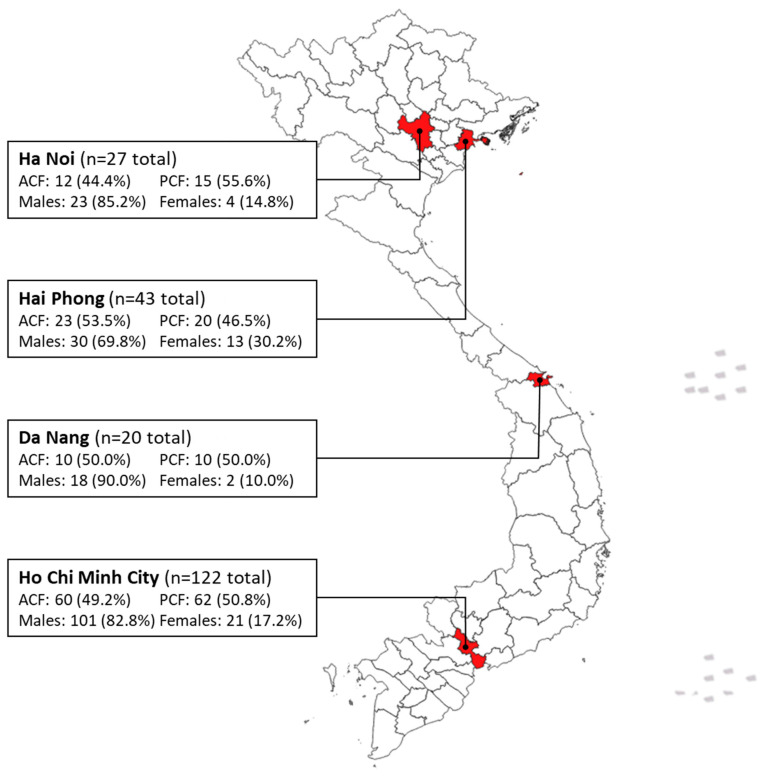
Map of this study’s sampling sites.

Study setting: Vietnam is a lower-middle-income country and one of the world’s 30 highest TB burden countries, with a TB incidence of 173/100,000 [3]. This study was implemented in four major cities located in the North, Central, and Southern areas of Vietnam: Ha Noi, Hai Phong, Da Nang, and HCMC (Figure 1). Community-based chest X-ray (CXR) screening events for TB were organized, and key populations, such as contacts, urban poor, economic migrants, diabetics, and older individuals (≥55 years), were mobilized. All ACF participants were screened by CXR, and those with abnormal findings were asked to provide a sputum sample for testing with the Xpert MTB/RIF Ultra assay [13].

Study population: Recruitment took place in the districts where ACF events were conducted; in these districts, a consecutive sampling approach was employed. Individuals diagnosed with TB at an ACF event and treated at the District TB Unit (DTU) were consecutively sampled across sites and referred for recruitment. We included people newly diagnosed with pulmonary, bacteriologically confirmed, drug-susceptible TB (DS-TB), aged 18 or above, residing in the district of treatment, who agreed to participate in this study. People with household members who were recruited for this study were excluded; only one person with TB per household was recruited. People with MDR-TB were excluded. Passively detected and treated individuals with TB were matched based on the treatment initiation date and residing district.

We estimated a sample size of 224 participants based on the catastrophic cost rate of 63% among DS-TB patients [10]. This estimate was powered to detect a 20% reduction in catastrophic cost incurrence with 95% confidence, 80% power, and 5% attrition [7,10].

Data collection: Information on monthly household and personal income, incurred costs, and time loss was collected using a locally adapted patient cost survey [9]. The survey also covered socio-economic consequences of TB, such as the use of coping strategies (loans and sale of assets), the experience of isolation, and loss of employment. To reduce recall bias, participants were longitudinally interviewed at three points during their treatment. Regular DS-TB treatment consisted of a two-month intensive phase, followed by a four-month continuation phase. Interviews were conducted at least two weeks into the treatment, at transition to continuation phase, and at the end of treatment.

Trained study officers administered interviews in person or via telephone according to COVID-19 guidelines. Participants produced receipts from all healthcare interactions at their second and third interview to reduce recall and social desirability bias. Data were collected on paper and via audio recording. Paper surveys were digitized using the Organizational Network Analysis data survey tool (Ona, NBO, Kenya). On a monthly basis, five percent of digital surveys were randomly selected and verified by another interviewer using the audio recordings.

Analysis: Baseline characteristics, costs, income, and catastrophic cost incurrence were tabulated by cohort. Categorical variables were expressed by frequencies/proportions and continuous variables by mean/standard deviation. Differences between survey cohorts were analyzed using chi-square, Fisher’s exact, or Wilcoxon rank-sum tests, as applicable.

Patient cost calculations followed WHO guidelines [9]. Direct medical costs included fees for consultations, diagnostic tests, hospitalization, and medication. Direct non-medical expenses included costs of food and travel arising from medical visits. Indirect costs refer to the reported income loss during treatment. To align with the national patient cost survey, we excluded caregiver time loss and calculated indirect cost using the output approach [10]. Direct medical, direct non-medical, and indirect costs were estimated for each treatment phase. Differences in costs and household income between ACF and PCF were compared using the Wilcoxon rank-sum test; differences across provinces were compared using the Kruskal–Wallis test. To explore potential associations between the case finding model, baseline characteristics, and catastrophic costs, we conducted both univariate and multivariate logistic regression analyses. The association between socio-economic consequences and the intervention was examined using chi-square tests. The shift in the proportion of households living in poverty (poverty headcount) between pre-TB and end of treatment was analyzed by means of McNemar’s test. We applied the World Bank poverty definition of a daily income of <USD 1.90 [14]. Monetary values were converted from VND to USD using the average conversion rate (VND 1 = USD 0.000043) during data collection [15]. Hypotheses were two-tailed, and *p*-values below 0.05 were considered statistically significant. Data analysis was performed using Stata v17 (Stata Corp, College Station, TX, USA).

Missing data: Only participants who completed all three interviews were included for analysis; there were no significant differences in age, sex, and household incomes pre-TB between complete and incomplete cases (n = 8). Missing household incomes prior to diagnosis were imputed using a declared household assets-based prediction. Data on household incomes at the time of the interview were complete. If the household income was lower than the personal income, we used the latter for the analysis. Missing personal income was imputed using the mean proportion of the household income of the whole sample.

## 3. Results

Table 1 describes the 212 study participants included in the analysis (105 ACF and 107 PCF). The majority of participants were men (81%), mean age was 54 years, and 83% were covered by social health insurance. At baseline, ACF participants were older (*p* < 0.001) and fewer had completed secondary school (*p* = 0.040). Those detected by ACF experienced symptoms for significantly less time before receiving treatment (4 vs. 6 weeks, *p* = 0.002) and had significantly fewer health provider visits (3 vs. 6 median visits, *p* < 0.001) compared with those passively diagnosed.

**Figure A1:**
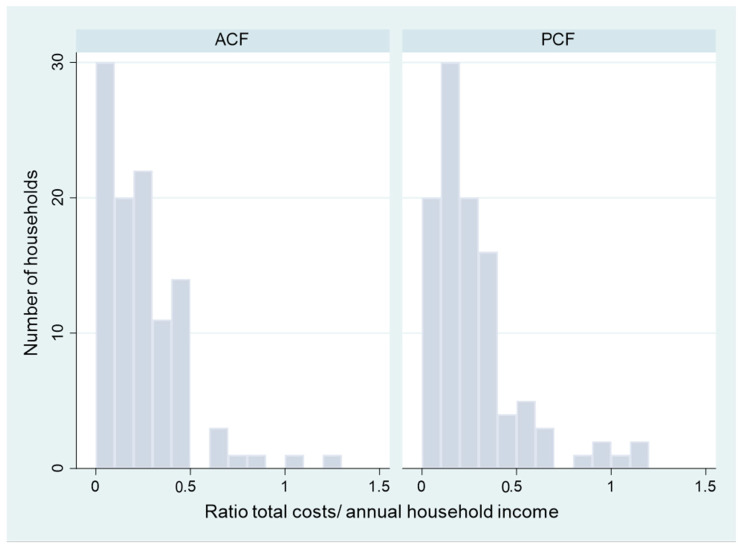
Comparison of total costs/annual household income ratio frequency in ACF and PCF cohort.

TB-related costs were high in both cohorts as illustrated in Table 2. For the total sample, the biggest contributor to total costs (pre-treatment and treatment) was indirect costs (USD 651) followed by direct non-medical (USD 236) and direct medical costs (USD 76). Total pre-treatment (USD 10 vs. 101, *p* < 0.001) and treatment costs (USD 888 vs. 1213, *p* = 0.043) were significantly lower in the ACF cohort. However, direct non-medical costs (USD 188 vs. 231, *p* = 0.294) and indirect costs (USD 468 vs. 925, *p* = 0.067) during treatment did not significantly differ. Both univariate and multivariate regression analyses found no significant associations between catastrophic costs and baseline characteristics or the case finding model (Table A1). ACF mainly reduced the total costs/income ratio of households falling below the 20% threshold (Figure A1).

**Figure 2:**
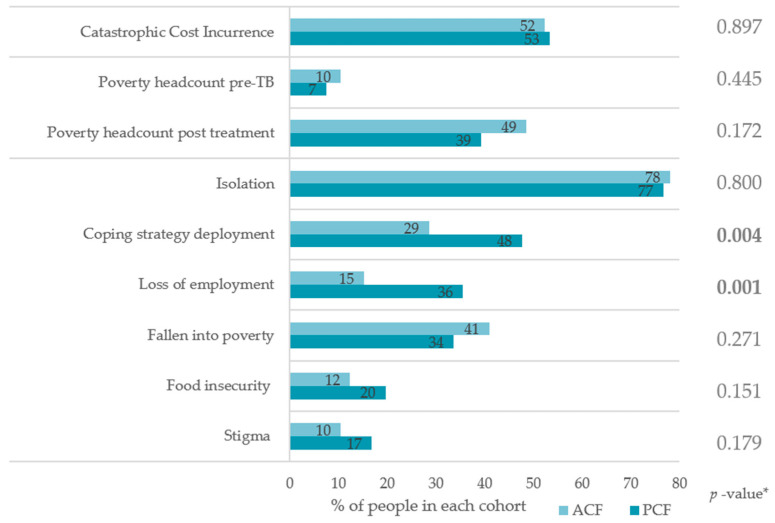
Comparison of catastrophic cost incurrence (total costs exceeding 20% of annual household income pre-TB) and socio-economic consequences; poverty line defined as personal income < USD 1.90/day; * chi-square test.

Catastrophic cost incurrence was high and comparable in both cohorts (52.4% vs. 53.3%, *p* = 0.897), as seen in Figure 2. The poverty headcount increased from 10.5% to 48.6% in the ACF (*p* < 0.001) and 7.5% to 39.3% in the PCF cohort (*p* < 0.001). There was no significant difference in people falling into poverty (41.0% vs. 33.6%, *p* = 0.271) between cohorts. Loss of employment (15.2% vs. 35.5%, *p* = 0.001) and the deployment of coping strategies (28.6 vs. 47.7%, *p* = 0.004) were significantly lower in the ACF cohort. Isolation, stigma, and food insecurity did not significantly differ.

**Figure 3:**
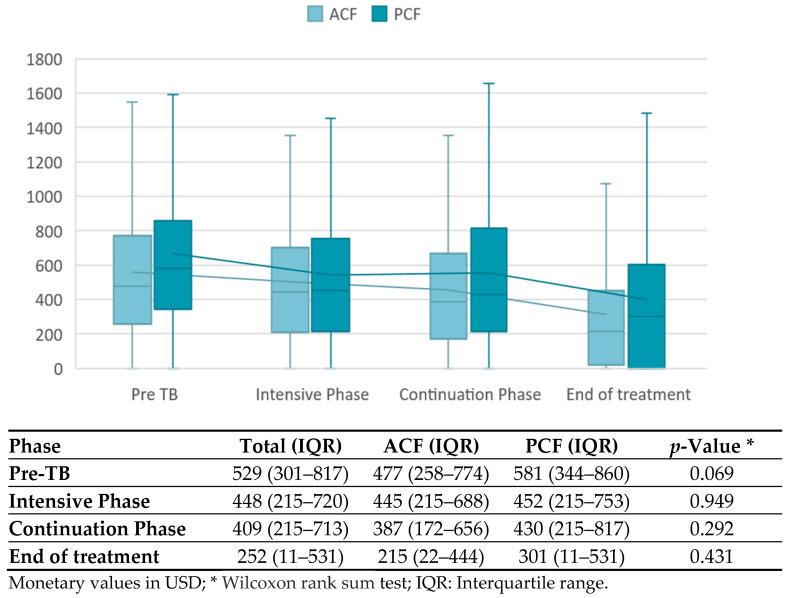
Median monthly household income (USD) over the course of treatment.

Figure 3 shows that household income did not significantly differ between ACF and PCF participants at any treatment phase. Over the course of treatment, incomes declined in both groups, from USD 477 to 215 in the ACF and USD 581 to 301 in the PCF cohort.

The cost differences between the cities are shown in Table 3. In HCMC, total direct costs were significantly lower (*p* < 0.001) and indirect costs higher (*p* < 0.001). The rate of catastrophic cost incurrence was highest in Hai Phong (60.5%), followed by Ha Noi (59.3%) and HCMC (54.1%), and was lowest in Da Nang (20.0%).

## 4. Discussion

Our study found TB-related pre-treatment and treatment costs to be significantly reduced in the ACF cohort. Moreover, this study revealed a decrease in rates of job loss and deployment of coping strategies in this cohort. Yet, no significant reduction in the proportion of participants detected via ACF who experienced catastrophic costs was detected.

Similar findings have been reported in Cambodia, India, and Nepal, where also no significant reduction in catastrophic cost incurrence was found [11,16,17]. After adjusting for confounders, some studies found a catastrophic cost risk reduction [12,18,19]. A similar analysis with our survey data did not change the findings from the crude analysis.

The significant reduction in pre-treatment costs in the ACF cohort is highly concordant with the present literature [11,12,16,17,18]. ACF also reduced total treatment costs indicating that early linkage to care may result in lower costs throughout treatment; such reductions in direct costs have been previously described [17,18,19]. Furthermore, a smaller proportion of participants in the ACF cohort suffered from loss of employment or resorted to coping strategies. Earlier diagnosis may reduce disease burden and thereby limit socio-economic consequences. Our study exhibited that those detected by active case finding made fewer health provider visits and were linked to treatment significantly quicker than those who were passively detected. Accelerated linkage to treatment can prevent progression of the disease and improve treatment outcomes, thereby reducing direct medical costs. We can conclude that, while ACF can contribute to the reduction in TB-associated cost burden, it is not enough to reduce catastrophic cost incurrence.

Similar to other studies, we have found that the largest driver of a participant’s catastrophic costs are indirect costs [8,11,12,17,18,19]. These were not reduced through ACF in our study. This was not surprising as participants in the ACF cohort received a one-off diagnostic intervention prior to TB treatment, with no additional support provided throughout their treatment follow-up. All people on TB treatment follow a mandated follow-up schedule and usually some level of dosing implementation monitoring/observation. This did not differ between the ACF and PCF cohorts.

In our study, household income continuously decreased over the course of treatment. A decrease in household income is often observed during the intensive phase of treatment [11,12,19]. A recovery to pre-treatment income at treatment completion was only observed in a previous ACF study in Vietnam, which took place prior to the COVID-19 pandemic [12]. In our study, recovery may not have taken place because of the economic consequences from lockdown restrictions enacted in response to the pandemic [20,21,22]. Households may remain economically vulnerable for months after treatment completion; therefore, more research on income trends following treatment completion is necessary to understand how TB-affected households can best be supported [23].

The ACF approaches may help in reaching the millions of people with TB who are currently missed and reduce their out-of-pocket costs, but alone, it will not achieve the End TB Strategy goals of zero catastrophic costs. Comprehensive social protection is clearly needed to reach global targets [24,25]. Similarly, universal health coverage alone is not enough, as it also does not address income losses [26]. To decrease the proportion of households experiencing catastrophic costs, protection from income losses is necessary. Social protection measures such as cash transfers have shown to decrease TB transmission, improve treatment outcomes, and reduce poverty in people with TB [27,28,29,30]. Currently, only a third of the Vietnamese labor force is part of the social insurance scheme [31]. Social protection measures should be combined with ACF to prevent income loss and catastrophic costs.

The use of dissaving strategies, which has previously been suggested as a possible proxy for catastrophic costs due to its association with total costs, was reported less frequently in the ACF cohort [32]. This does not reflect literature from Nepal and Cambodia, where no significant differences were found, and Vietnam, where the opposite had been previously observed despite lower total costs in the ACF participants [12,16,17,19]. Therefore, one should be cautious with the use of coping strategies as a marker for financial hardship. A decreased rate of loss of employment in the ACF cohort was also not observed in other studies. Interestingly, social exclusion/isolation was reported in no more than a quarter of the study populations, while being the most frequently reported consequence in our study [11,12,19]. The COVID-19 pandemic, and consequently social distancing, may be the cause for this difference.

Our population was similar to the national patient cost survey in regard to age, education, and sex; nonetheless, catastrophic cost incurrence in our study was lower [10]. A possible reason is that the national survey included people with multidrug-resistant TB, who are known to face a larger financial burden [8]. The median pre-treatment household income in our study was higher (USD 529), likely due to the rural sample population in the national survey (USD 322); it was similar to previous TB patient cost analyses in HCMC (USD 483–503) [12,22]. We found large disparities in costs across cities. Due to the limited sample size, these differences were not further analyzed. Different practices in hospitalization and decentralization of patient follow-up may contribute to the costs. More research is needed to understand the drivers of higher costs between regions. Despite 83% of people being insured under Vietnamese Social Health Insurance and free of charge TB medication, substantial direct medical costs were experienced. Strategies on how to decrease medical costs further warrant investigation.

### Strengths and Limitations

At the time of publication, our study is the largest to compare ACF and PCF in Vietnam, including people with TB from the four large cities. The analysis encompasses patient costs, as well as the socio-economic consequences of TB. The usage of the WHO patient costing survey tool enables the comparison with other settings. The longitudinal design improves the validity of findings and minimizes recall bias.

There were some differences in baseline characteristics between the cohorts, but since ACF focuses on vulnerable populations, we saw this as an accurate representation of reality and hence chose not to correct for it. Generalizability is limited due to the sample size. Some clear tendencies were not statistically significant indicating limited power. Lastly, data collection took place amid the COVID-19 pandemic, which decreased the household incomes in Vietnam, resulting in greater income losses and potentially preventing observable income recovery [20,21,22].

## 5. Conclusions

In our study, ACF significantly decreased TB-related costs and negative socio-economic consequences. However, it did not suffice to reduce catastrophic cost incurrence in people with TB. This underscores the limitations of solely relying on ACF. Social protection mechanisms are needed to mitigate indirect costs, the largest contributor to catastrophic costs.

## Figures and Tables

**Table 1:** Baseline participant characteristics from the ACF and PCF cohorts.

	All N = 212	ACF N = 105	PCF N = 107	*p*-Value *
Men, N (%)	172 (81.1%)	85 (80.9%)	87 (81.3%)	0.947
Age, mean (SD)	54 (16)	62 (12)	47 (15)	**<0.001**
Age group, N (%)				**<0.001**
18–24	11	0	11	
25–34	22	3	19	
35–44	20	5	15	
45–54	42	20	22	
55–64	64	40	24	
65+	53	37	16	
Completed secondary school, N (%)	60 (28.3%)	23 (21.9%)	35 (34.6%)	**0.040**
Employment (including informal) pre-TB, N (%)	191 (90.1%)	95 (90.5%)	96 (89.7%)	0.854
Monthly household income pre-TB in USD, median (IQR)	529 (301–817)	477 (258–774)	581 (344–860)	0.069
Households living below poverty line **, N (%)	19 (9%)	11 (10%)	8 (7%)	**0.445**
Number of household members, mean (SD)	4 (2)	4 (2)	4 (2)	0.061
Enrolled in Social Health Insurance, N (%)	176 (83.0%)	88 (83.8%)	88 (82.2%)	0.761
Diagnosed with at least one comorbidity, N (%)	151 (71.2%)	75 (71.4%)	76 (71.0%)	0.949
History of TB, N (%)	12 (5.7%)	8 (7.6%)	4 (3.7%)	0.250
Self-reported positive HIV status, N (%)	5 (2.4%)	0 (0.0%)	5 (4.7%)	0.060
Weeks before treatment, median (IQR)	5 (1–15)	4 (0–12)	6 (3–15)	**0.002**
Health provider visits before diagnosis, median (IQR)	4 (3–7)	3 (2–4)	6 (3–7)	**<0.001**

* Chi-square test/Fisher’s exact test/Wilcoxon rank-sum tests as applicable; ** poverty line defined as personal income < USD 1.90/day; SD = standard deviation; significant *p*-values marked in bold.

**Table 2:** TB costs in USD at different stages of treatment.

	All (N = 212)		ACF (N = 105)		PCF (N = 107)		
	Mean (SD)	Median (IQR)	Mean (SD)	Median (IQR)	Mean (SD)	Median (IQR)	*p*-Value *
Total cost (pre-treatment and treatment)							
Direct medical	201 (374)	76 (17–226)	118 (269)	21 (6–76)	282 (440)	141 (75–292)	**<0.001**
Direct non-medical	338 (442)	236 (106–431)	298 (337)	200 (82–391)	376 (524)	304 (136–448)	0.095
*Total direct*	536 (683)	350 (151–668)	416 (555)	258 (108–483)	655 (773)	429 (250–701)	**<0.001**
Indirect	1052 (1292)	651 (12–1485)	979 (1497)	468 (0–1328)	1124 (1147)	925 (75–1559)	0.067
*Total treatment*	1588 (1466)	1208 (520–218)	1395 (1498)	957 (361–1862)	1779 (1415)	1359 (724–2482)	**0.006**
Pre-Treatment							
Direct medical	113 (276)	30 (3–97)	39 (118)	6 (0–20)	186 (356)	82 (40–164)	**<0.001**
Direct non-medical	28 (72)	4 (1–23)	12 (37)	2 (1–5)	51 (153)	10 (1–26)	**<0.000**
*Total direct*	141 (328)	46 (5–143)	51 (153)	10 (1–26)	229 (420)	101 (56–198)	**<0.000**
Treatment							
Direct medical	87 (212)	13 (0–78)	79 (234)	7 (0–34)	96 (188)	35 (0–105)	**0.025**
Direct non-medical	310 (426)	218 (85–396)	286 (332)	188 (66–356)	334 (502)	231 (95–419)	0.294
*Total direct*	395 (542)	245 (102–540)	365 (528)	224 (81–409)	425 (556)	276 (115–591)	0.141
*Total treatment*	1449 (1393)	1088 (477–2041)	1343 (1497)	888 (317–1693)	1553 (1282)	1213 (649–2153)	**0.043**

Direct medical costs: fees for consultations, diagnostic tests, hospitalizations, and medication; direct non-medical costs: food, travel, and accommodation costs; indirect costs: income loss; monetary values in USD; * Wilcoxon rank-sum test (medians); SD: standard deviation; IQR: interquartile range; significant *p*-values marked in bold.

**Table 3:** Comparison of costs across different cities.

	Total (n = 212)	Ha Noi (n = 27)	Hai Phong (n = 43)	Da Nang (n = 20)	HCMC (n = 122)	*p*-Value *
Household income pre-TB						
Median monthly income (IQR)	529 (301–817)	473 (301–718)	505 (237–688)	645 (241–871)	581 (505–237)	0.106
Pre-Treatment costs						
Median direct costs (IQR)	30 (3–97)	46 (12–93)	91 (9–160)	13 (0–42)	21 (0–84)	**0.008**
Treatment costs						
Median direct costs (IQR)	245 (102–540)	550 (196–847)	372 (224–696)	257 (205–334)	181 (54–356)	**<0.001**
Median indirect costs (IQR)	651 (16–1485)	344 (0–1134)	169 (0–1333)	16 (0–778)	1066 (301–1832)	**<0.001**
Median total costs (IQR)	1088 (477–2041)	979 (587–2131)	823 (307–1806)	668 (249–988)	1249 (527–2266)	**0.033**
Total costs (pre-treatment and treatment)						
Median direct costs (IQR)	350 (151–668)	602 (272–959)	517 (300–980)	317 (219–398)	227 (110–494)	**<0.001**
Median indirect costs (IQR)	651 (16–1485)	344 (0–1134)	169 (0–1333)	16 (0–778)	1066 (301–1832)	**<0.001**
Median total costs (IQR)	1208 (520–2189)	1125 (587–2328)	1152 (398–1932)	668 (258–1000)	1360 (591–2470)	**0.020**
Catastrophic cost incurrence						
Number of households (%)	122 (52.8%)	16 (59.3%)	26 (60.5%)	4 (20.0%)	66 (54.1%)	**0.016** °

Monetary values in USD; * Kruskal–Wallis test; ° Fisher’s exact test; significant *p*-values marked in bold.

## Data Availability

Study protocols and statistical analysis are available upon reasonable request from the corresponding author, AMCW. The data that support the findings of this study are not publicly available due to containing information that can compromise the privacy of research participants. Anonymized data can be made available upon reasonable request and with approval from the Ha Noi Lung Hospital, Pham Ngoc Thach Hospital, Hai Phong Lung Hospital, and Da Nang Lung Hospital.

## Appendix A

**Table A1:** Association between baseline characteristics and catastrophic cost incurrence.

Baseline Characteristic	Catastrophic Cost Incurrence, N (%)	Crude OR * (95% CI)	Adjusted OR ** (95% CI)
*Case finding model*			
PCF	55/105 (52.4%)	1.00	1.00
ACF	57/107 (53.3%)	0.96 (0.56–1.65)	0.93 (0.52–1.69)
*Sex*			
Men	89/172 (51.7%)	1.00	1.00
Women	23/40 (57.5%)	1.26 (0.63–2.53)	1.27 (0.62–2.57)
*Age category*			
≤50 years	38/73 (52.1%)	1.00	1.00
>50 years	74/139 (53.2%)	1.05 (0.59–1.85)	1.10 (0.56–2.00)
*Education level*			
Up to primary education	55/92 (59.8%)	1.00	1.00
Secondary school or above	57/120 (47.5%)	0.61 (0.35–1.05)	0.60 (0.34–1.07)
*Employment status*			
Unemployed	102/191 (53.4%)	1.00	1.00
Employed	10/21 (47.6%)	1.26 (0.51–3.11)	1.41 (0.56–3.56)
*Social Health Insurance status*			
Uninsured	21/36 (58.3%)	1.00	1.00
Insured	91/176 (51.7%)	0.76 (0.37–1.58)	0.84 (0.39–1.78)

* Univariate logistic regression; ** multivariate logistic regression incorporating all shown patient covariates as exposure parameters for the model; CI = confidence interval.
